# A mini-screen to identify the role of microRNAs in the Drosophila Insulin-Producing cells regulating lifespan

**DOI:** 10.17912/micropub.biology.000536

**Published:** 2022-03-17

**Authors:** Jervis Fernandes, Jishy Varghese

**Affiliations:** 1 School of Biology, Indian Institute of Science Education and Research Thiruvananthapuram (IISER TVM), Thiruvananthapuram, Kerala, India 695551

## Abstract

Various genetic, molecular and environmental factors influence the lifespan of an organism, which includes the highly conserved insulin signaling pathway. In *Drosophila* , Insulin-Producing cells (IPCs) present in the fly brain, analogous to vertebrate pancreas, control growth, metabolism and lifespan. Regulation of gene expression by microRNAs is strongly believed to be crucial in determining adult lifespan, however, no systematic approach has been conducted so far to study the role of microRNAs in the IPCs in the aging process. Most of the current work has been focused on deciphering the roles of microRNAs in the IPCs during developmental stages. Here, we report the results of a mini-screen performed to identify microRNAs that function in the adult IPCs in regulating lifespan and neuronal integrity.

**Figure 1. Role of microRNAs in the Insulin-producing cells in regulation of lifespan and climbing ability:**
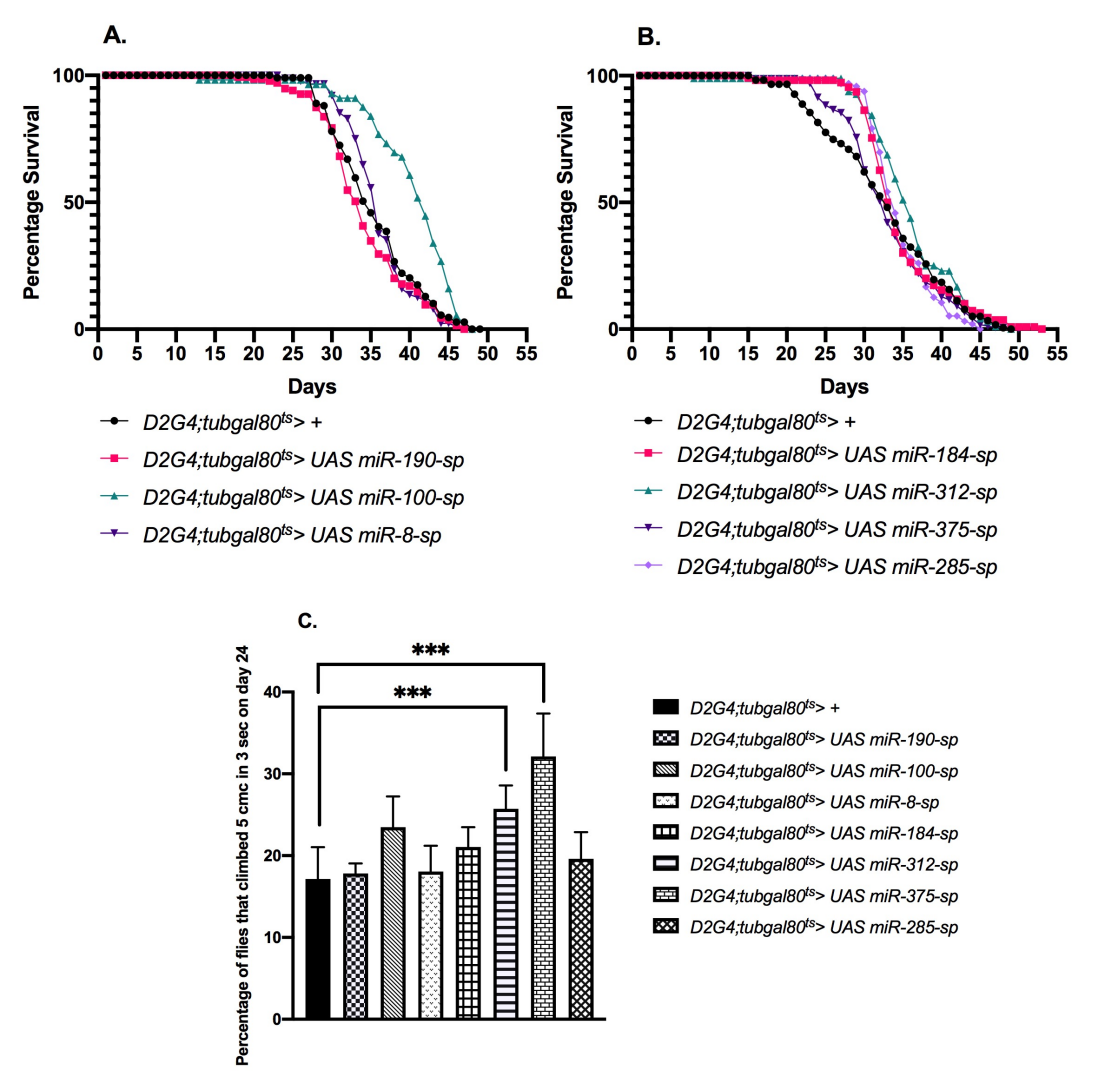
Survivorship curves upon expression of miR-100, mIR-190, miR-8, miR-184, mIR-312, miR-375 and miR-285 sponges in adult IPCs (A-B). An increase in lifespan was observed when miR-100 sponge was expressed in the adult IPCs (A). An increase in survival in early lifespan was observed when miR-312 sponge and miR-285 sponge were expressed in the adult IPCs (B). Percentage flies crossing 5 cm in 3 secs upon expression of miR-100, miR-190, miR-8, miR-184, miR-312, miR-375 and miR-285 sponges in adult IPCs on day 24 (C ). An increase in climbing ability on day 24 was seen upon expression of miR-312 sponge and miR-375 sponge in the adult IPCs (C ). n ≥ 56. Log-rank test (A-B), Fleming-Harrington test (A-B) and a student's t-test were performed to test statistical significance (*, *p<0.05;* **, p <0.01; ***, p<0.001; n.s., not significant). *D2G4* is an abbreviation for *dilp2Gal4.*

## Description

The lifespan of organisms is controlled by both genetic and environmental factors. Nutrition is one of the key environmental factors that regulates the lifespan of an organism (Kwang et al. 2008). Among the molecular pathways, insulin signaling (Bartke 2008; Schuh et al. 2011; Tatar et al. 2001), TOR signaling (Kapahi et al. 2004) and JNK (c-Jun N-terminal Kinase) signaling (Wang, Bohmann, and Jasper 2005) have been reported to affect lifespan. Various other factors like oxidative stress (Liochev 2013), metabolic status, telomere length (Armanios et al. 2009; Vera et al. 2012), and DNA damage (Flurkey, Currer, and Harrison 2007) have previously been implicated to regulate lifespan in mammals.

The insulin/insulin-like growth factor (IGF) pathway links nutritional status to growth, metabolism and lifespan. In *Drosophila* , 8 insulin-like peptides (DILPs), similar to vertebrate insulin/IGFs have been discovered to date. Of which DILP2, 3 and 5 are produced by the insulin-producing cells (IPCs) located in the *pars intercerebralis* region of the *Drosophila* brain. In addition to the IPC DILPs, DILP6 is produced from the fatbody, DILP7 from the intestine and DILP8 from the imaginal discs and ovaries (Semaniuk et al. 2021).

Earlier studies have demonstrated an increase in lifespan in flies that are defective for insulin signaling (Clancy et al. 2001; Tatar et al. 2001). Ablation of the IPCs was also found to increase the lifespan (Broughton et al. 2005; Min et al. 2008). A significant increase in longevity was observed in *dilp2* mutants (Grönke et al. 2010). Increased lifespan caused by dietary restriction is mediated by the neuronal DILPs (Piper, Skorupa, and Partridge 2005). Overexpression of *dilp6* in the adult head and abdominal fatbody, has also been demonstrated to increase longevity, possibly through the regulation of *dilp2* (Bai, Kang, and Tatar 2012). Recent data also suggests that *dilp1* could play a role in regulating lifespan (Post et al. 2019). In a recent study, age-related decrease in *dilp3* levels with age was implicated to be responsible for Intermediate-Term Memory Impairment in *Drosophila* (Tanabe, Itoh, and Tonoki 2017).

microRNAs are short non-coding RNAs that post-transcriptionally regulate the levels of various target mRNAs that they bind to via sequence complementarity (Ambros 2001). microRNAs are known to either regulate insulin levels directly or regulate insulin signaling in the responsive tissues. miR-8 in *Drosophila* , was found to promote insulin signaling and body growth, by regulating its target genes, *u-shaped* ( *ush* ), and *dilp6* , in the fatbody (Hyun et al. 2009). *miR-278* mutants were found to be insulin resistant and that miR-278 acts through regulation of the *expanded* transcript (Teleman, Maitra, and Cohen 2006).

Quite a few microRNAs have been discovered to regulate the levels of insulin-like peptides directly. miR-14 has previously been discovered to regulate the insulin-like peptides and thereby the metabolism via its target *sugarbabe* (Varghese, Lim, and Cohen 2010). miR-11 has been shown to regulate pupal size via its target RAS85D by regulating the gene expression of *ilp2, 3* and *5* from the IPCs (Li et al. 2016). miR-7 regulates the production and secretion of insulin-like peptides from the IPCs via its target, *Drosophila* *F-actin capping protein alpha* (Agbu et al. 2020). A large genetic screen, identified that the conserved miR-9a regulates body growth by targeting sNPFR1 in the IPCs in *Drosophila* (Suh et al. 2015).

Recently various studies have been carried out to estimate the levels of all microRNAs in the fly brain/head to discover any role that microRNAs could play in aging. The level of miR-34 was found to go up in the head of flies with age and thus regulate lifespan via its target *Eip74E* (Liu et al. 2012) *.* An age-dependent increase in all the three let-7-C miRNAs in both adult males and females flies, was observed (Chawla et al. 2016a). All of this work suggests the possibility that the level of various microRNAs in each cell type or cell could be dynamic and change in an age-dependent manner as well.

Adult specific overexpression of miR-184 and let-7 led to significant changes to fat metabolism and life span in Drosophila (Gendron and Pletcher. 2017). Both let-7 and miR-125 were found to regulate *chinmo* and thereby regulate lifespan (Chawla et al. 2016a). miR-969 (Zhang et al. 2020), miR-305 (Ueda et al. 2018), miR-277 (Esslinger et al. 2013) and miR-282 (Vilmos et al. 2013) independently were also found to play a role in the regulation of lifespan in *Drosophila* . Few microRNAs were discovered to circulate in the hemolymph of *Drosophila* and were found to accumulate in tissues in an age-dependent manner (Dhahbi et al. 2016). Thus highlighting the importance of studying the role any microRNAs may play in regulating lifespan.

Here, we carried out a mini-genetic screen to identify microRNAs which when downregulated in the IPCs play a role in regulating lifespan. We analyzed the effects of downregulation of 7 conserved microRNAs which are known to be expressed in the fly head. The screen was carried out by expressing microRNA-sponges (Fulga et al. 2015) specifically in the Insulin-producing cells in the adult male *Drosophila* with the use of a *dilp2GAL4; tubulinGAL80 ^ts^* line. This approach was taken to ensure that the effects seen are not caused by reducing the levels of these microRNAs during stages prior to adulthood. We also carried out a climbing assay on day 24, as a readout for neuronal integrity in response to aging in these flies.

A significant increase in lifespan was observed when miR-100 sponge was expressed in the IPCs of adult male flies (Figure 1.A). In a prior study, miR-100 levels were found to increase with age (Chawla et al. 2016b). Therefore it would be interesting to verify any role that the microRNA may play in regulating DILPs from the IPCs in adult flies. To check for any early or late changes in death, we also carried out a Fleming-Harrington weighted log rank-test Test wherein, flies started dying later when miR-312 sponge (p=0.0294) and miR-285 (p=0.0459) sponge was expressed in the IPCs of adult male flies, without affecting the overall lifespan (Figure 1.B). Expression of miR-184 sponge in the IPCs led to a similar but not statistically significant increase in survival during early lifespan (p=0.0567). None of the other microRNA sponges tested showed any effects on lifespan. The climbing ability of flies in response to expression of miR-375 (p=0.00653) and miR-312 sponges (p=0.00656) in the IPCs was found to be significantly higher than that of the control (Figure 1.C). Expression of miR-100 sponge in the adult IPCs also improved the climbing capacity of older flies (though this was not statistically significant) (Figure 1.C). In a screen carried out previously, overexpression of miR-312 and miR-285 in the IPCs led to an increase in wing size (Suh et al. 2015). Further experiments are needed to verify the role that miR-100, miR-375, miR-285 and miR-312 play in the IPCs in neuronal function and aging.

## Methods

Survival assay

Larvae were raised on standard media at 18 ^O^ C, flies were allowed to mate at 29 ^O^ C for two days. Batches of 10-15 male flies per vial were provided fresh food every alternate day with dead flies being recorded every day. A Log rank test and a weighted-log rank test with a Fleming-Harrington test were performed using the OASIS 2 software.

Climbing assay

Climbing assay was carried out using long vials. Flies were transferred to assay vials and acclimatised for 10 minutes, the vial was tapped thrice in an arrhythmic fashion, and the number of flies that cross 5 cms in 3 seconds were noted. This assay was carried out thrice with a gap of about 3 minutes between each trial. A student's t-test was carried out to calculate if there were any significant changes if any.

Statistical analysis

Prism 8 was used to plot all the data. OASIS 2 (Han et al. 2016) was used for the statistical analysis for the lifespan experiments. A log-rank test was carried out to check for differences in lifespan while a Fleming-Harrington test was carried out to check for differences in early or late survival. The percentage of flies crossed 5 cms in 3 secs was calculated. The percentages were compared with the control using a student's t-test.

## Reagents

Fly stocks

*dilp2Gal4* is described in Ikeya et al. 2002, *tubulin-Gal80 ^ts^* (7017), and *w ^1118^* (5905) are originally from Bloomington Drosophila Stock Center.

**Table:** 

**Fly line**	**Genotype**	**Bloomington stock number**
miR-190-sp	w[*];P{y[+t7.7]w[+mC]=UAS-mCherry.mir-190.sponge.V2}attP40;P{y[+t7.7]w[+mC]=UAS-mCherry.mir-190.sponge.V2}attP2	61397
miR-100-sp	w[*];P{y[+t7.7]w[+mC]=UAS-mCherry.mir-100.sponge.V2}attP40/CyO;P{y[+t7.7]w[+mC]=UAS-mCherry.mir-100.sponge.V2}attP2	61391
miR-8-sp	w[*];P{y[+t7.7]w[+mC]=UAS-mCherry.mir-8.sponge.V2}attP40/CyO;P{y[+t7.7]w[+mC]=UAS-mCherry.mir-8.sponge.V2}attP2	61374
miR-184-sp	w[*];P{y[+t7.7]w[+mC]=UAS-mCherry.mir-184.sponge.V2}attP40;P{y[+t7.7]w[+mC]=UAS-mCherry.mir-184.sponge.V2}attP2/TM6B, Tb[1]	61396
miR-312-sp	w[*];P{y[+t7.7]w[+mC]=UAS-mCherry.mir-312.sponge.V2}attP40/CyO;P{y[+t7.7]w[+mC]=UAS-mCherry.mir-312.sponge.V2}attP2	61429
miR-375-sp	w[*];P{y[+t7.7]w[+mC]=UAS-mCherry.mir-375.sponge.V2}attP40;P{y[+t7.7]w[+mC]=UAS-mCherry.mir-375.sponge.V2}attP2	61436
miR-285-sp	w[*];P{y[+t7.7]w[+mC]=UAS-mCherry.mir-285.sponge.V2}attP40/CyO;P{y[+t7.7]w[+mC]=UAS-mCherry.mir-285.sponge.V2}attP2	61417
